# Recent advancements in machine vision methods for product code recognition: A systematic review

**DOI:** 10.12688/f1000research.124796.1

**Published:** 2022-09-27

**Authors:** Jarmo Koponen, Keijo Haataja, Pekka Toivanen

**Affiliations:** 1 School of Computing, Kuopio campus, University of Eastern Finland, Kuopio, Pohjois-Savo, FI-70211, Finland

**Keywords:** Machine Vision, Imaging System, Character Recognition, OCR, Deep Learning, Product, Packaging, Manufacturing

## Abstract

**Background:** Manufacturing markings printed on products play an important role in the handling and use of pharmaceuticals and perishable foods. Currently, optical character recognition, neural networks, deep learning-based methods, and combinations of these methods are used to recognize these codes.

**Methods:** This systematic review was performed to find papers that can answer the following research questions: How have machine vision methods that can recognize product texts evolved over the past eight years? What are the most common difficulties in recognizing product texts? Articles published between 2012 and 2020 were systematically searched from Science Direct/SCOPUS, and Google Scholar in November-December 2020. Ten studies were eligible, with inclusion criteria: details about the recognition method used, performance analysis result, imaging method, product and the text printed on it.

**Results:** Product text recognition methods have evolved significantly over the last two years to tolerate the most common difficulties in the field. This is due to the ability of the deep learning neural network (DNN) architectures such as convolutional neural networks (CNN) to extract and learn salient character features straight from packaging surface images. Four of the most recent methods use two consecutive deep learning networks, one detecting the text area based on an image captured from the product package's surface and the other recognizing the characters within. Furthermore, this paper presents solutions to the most common product text recognition difficulties.

**Conclusions:** There were a limited number of studies that met the eligibility criteria for this systematic review. The study's aim was to evaluate the development of machine vision methods for recognizing manufacturing marking texts printed on the surface of products. The study results demonstrated how methods have evolved over time, beginning with optical character recognition, and advancing to methods which can recognize texts despite the field's most common problems.

## Introduction

The reported value of food and pharmaceutical production sold in the European Union (EU) in 2020 was over €105 billion and over €30 billion respectively. ^1^ The manufactured product moves through the supply chain from the factory to its end users. The product requires primary packaging for its handling, secondary packaging may be required for storage, and tertiary packaging for its transport. ^2^ The packaging has multiple functions, to protect the product during handling, storage, and transport, as well as prevent contamination and spoilage of the product. ^3^ On the other hand, it should increase the usefulness and convey information for the many uses of the product. ^2^^,^^3^ Production information, such as batch code, serial number, or expiration date, is printed on specific fields of the product, or its package at the manufacturing stage. Each of these consists of numbers and letters with a defined structure and length.

One key point in EU food safety legislation is the ability to trace products throughout the food chain. ^4^ All perishable food products intended for human consumption must be marked with the date which their consumption is no longer considered safe. ^5^ Likewise, finished medicinal products must be identifiable by the labels required by national law, including the expiry date in uncoded form and the batch number provided by the manufacturer. ^6^

However, even today, the traditionally necessary step to check the expiration date information printed on packages is done by a human operator who manually picks up the package and checks the date. This is an everyday, monotonous, and high precision task, placing the human in an error-prone working environment. ^7^ Instead of a human operator, machine vision could be used for product codes text recognition without contact with higher accuracy and speed.

Converting these product codes into a machine-coded format is more difficult than optical text recognition of paper documents. However, it enables storage and processing of package-specific production data as well as search and extraction of codes and dates electronically.

Existing optical character recognition (OCR) techniques work effectively only with clear characters in high-quality images on an uncomplicated background, while requiring character consistency in terms of format and viewing angle. ^7^ Common problems in recognizing the texts are caused by the complexity and deformation in the facet where the codes are printed, its problematic illumination, rich color information, insufficient contrast of the text, different printing types, and inconsistency of text characters. ^5^^,^^7^^–^^10^ Although the package is regular in shape after the product has been manufactured, character distortion and warping during the storage duration can cause challenges for traditional OCR. ^9^ Changes in physical conditions, such as different sizes and shapes of product packages, different positions, and angle of placement of the package in the camera view, and work environments lighting conditions make text recognition more difficult. When capturing images of packets moving on a conveyor belt, due to the motion blur caused by speed, the images may be blurred, making the recognition more difficult. ^11^ The recognition of product codes from natural images is still a challenging task, as images may contain text with arbitrary perspective deformations in a complex background due to its unknown 3D position and orientation. ^7^^,^^11^

Neural networks modeled from brain structure are today very widely used in text recognition applications that require complex and large numbers of feature classification capabilities. At the same time, the graphics cards required for model training of neural networks are constantly evolving in terms of computing power and storage capacity. Several researchers propose neural network-based methods for detecting and recognizing packaging texts in recent studies. ^7^^,^^10^^,^^11^ However, training in the deep neural network (DNN) model required familiarity with the subject and a large number of experiments to determine what is a viable way to solve the text recognition problem in question.

In recent years, machine vision technology has been increasingly used in a wide range of product codes recognition: it is used in the design of sustainable food systems to reduce food waste, ^12^^,^^13^ improving safety in the use of perishable food and pharmaceutical products, ^4^^,^^9^ and in developing faster and more accurate methods for the retail supply chain. ^5^^,^^14^^,^^15^ Electronic processing of product-specific serial numbers is also suitable for electronic inventory management systems, ^16^ development of intelligent product code recognition systems to help the daily lives of visually impaired people, ^10^ development of intelligent household refrigerator food management systems, ^17^ and also for the needs of metal industry to automatically verify the serial numbers of metal products at different stages of production. ^18^

**Figure 1.**
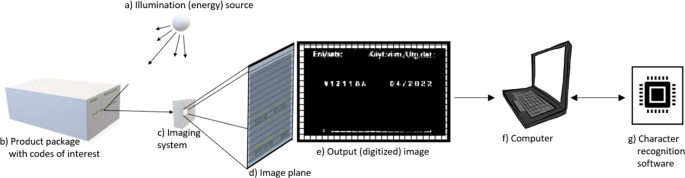
An example of the imaging and character recognition process of product packaging. (a) Energy source. (b) Product package. (c) Imaging system. (d) Projection of the scene onto image plane. (e) Digitized image. (f) Computer. (g) Character recognition software.

For individual photosensitive sensors used in digital imaging, the dominant arrangement is a 2D array form. The imaging system (see Figure 1c) collects the incoming energy and focus it onto the image plane (see Figure 1d). In bright illumination lens at the front end of the imaging system projects the scene being viewed onto the lens focal plane. The sensor array is coincident with the focal plane, it produces output proportional to the integral of the light received at each sensor. Electronic circuits sample the outputs, and another part of the imaging system digitizes the signal and produces an output image (see Figure 1e). ^19^

With good illumination, the imaging system captures a clear image of the flat surface of the packages. The computer (see Figure 1f) and its character recognition software (see Figure 1g) are used to recognize the codes printed on it. Regularly shaped characters with good contrast against a simple background can be recognized with OCR software. ^20^ Imperfections in the orientation of the target surface, in its flatness, in the contrast of the text, in the consistency of the characters, or in the regularity of the printed text on the packaging surface complicates the recognition task. In addition to OCR, ^21^ the subject of product code recognition has been approached in a number of methods. To extract representative features of characters, binary large object detection (BLOB), ^16^ Histograms of Oriented Gradients (HOG), ^18^ and Gabor-filtering ^8^^,^^9^ have been utilized, whereas classifiers and neural networks have been used to categorize them. Recently, the topic has been addressed utilizing end-to-end execution with Deep learning, such as fully Convolutional Neural Network, Mask R-CNN neural network, and Connectionist Text Proposal Neural Network (CTPN), for text region detection and recognition. ^7^^,^^10^^,^^11^^,^^15^

This research aims to advance the knowledge base in developing smart product handling methods for future researchers by:

• Providing detailed knowledge of current algorithms for text recognition methods for product codes.
• Systematically analyzing and presenting an overview of machine vision- based product code recognition techniques for each stage, (Code characters: extraction, segmentation when applicable, and recognition), with a brief description of the techniques used in each stage.
• Summarizing the performance of algorithms developed by various researchers and used and tested for product code recognition.
• Providing knowledge of the associated imaging environment used and tested for product code recognition, with details on the surface properties of product packaging and printed texts.

## Methods

The systematic review method used in this study can be considered as somewhere between the traditional and meta-analysis review approaches. This method has been chosen to reduce the risk of bias and increase reliability. The evaluation was conducted in accordance with the guidelines known as PRISMA statement [Reporting guidelines].

This work examines the literature to answer the following research questions:

• How have machine vision methods capable of recognizing product texts evolved over the last eight years?
• What are the most common difficulties in recognizing product texts?

Answers are sought by formulating a research question and collecting data from a scholarly database, primarily Scopus, and secondly searching for related publications in the Google Scholar database. The search strategy for Scopus used in this study was a string of characters composed specifically of the following terms: batch code, batch, expiration date, expiry date, serial number, manufacturing, OCR, machine vision, computer vision, detection, recognition, and combinations thereof. The search was limited to literature published during the last eight years (2012-2020). Publications related to manufacturing, retail chain, and serial production were retrieved from the Google Scholar. The following criteria were used to select the appropriate studies to review:

• The studies which include predefined keywords in the article title, abstract or keywords.
• Those who deal with product codes text recognition which are based on computer vision.
• Those that provided details of the performance analysis with details of the imaging method, product, printed text, and the method of text recognition itself.
• Those studies that were published between 2012 and 2020.
• Those that can be accessible.

The following criteria were used to exclude the studies from the scope of review:

• The studies which are not written in English.
• The studies with unidentified reference.
• The studies published before the year 2012.

In total, 138 articles were extracted. The following is a detailed analysis of the product text recognition methods used in the 10 studies, as well as the recognition results achieved with them.

### Conventional recognition methods

According to the Althobaiti *et al.,* ^20^ OCR is the process of converting text in images into a machine-coded form. The first step in OCR is to find the optical characters in the input image. OCR works by collecting detected dots (pixels) from an image, which are then compared to a model taught to the system. This is used to identify a detected character, which can be a letter, number, or special character. If characters form a group, this group is compared with possible grammatical words, and the correctness of the recognition can be automatically concluded. An OCR- based expiration date recognition system for the visually impaired is described in Peng *et al.* ^21^ It recognizes date codes of consumer products using cell phone camera and guides the user with voice feedback. This method works in two steps: First, the product barcode is detected. Next, the date code location information for the corresponding available facet and surface area is retrieved from the database and the date code is recognized therefrom by OCR. The accuracy of the proposed method reached 100% in all tests, while it is only 10-20% for the baseline system, which detects date-like texts from the wrong range and often misses the expiration date completely due to missing text location information.

Gabor energy response based expiration code detection and recognition method for food packaging has been proposed in Zaafouri *et al.* ^8^ Method use source images captured from the packages with a standard digital camera. The expiration code is localized based on the local energy calculation of the images, the determination of the maximum energy difference, and the analysis of connected components. Characters found are binarized, and further segmented characters are convolved with a bank of Gabor -filters to extract three Gabor features: Fourier magnitude, imaginary response, and Gabor energy- response. Characters are classified by a sparse representation-based classifier using the Gabor energy -response. The method was tested with different backgrounds, code directions, and contrasts. It consistently located codes from images but suffered limitations with complex backgrounds and when the characters composing the code derived from location and isolation modules are very distorted. Moreover, it is sensitive to parameter selection, especially Gabor filter parameter settings. Furthermore, comparing the execution time of the proposed algorithm of 4.6 s with the execution time of the edge-based algorithm of 2.1 s in the corresponding task shows the slowness of the method.

A method based on Stretched Gabor features was proposed in Zaafouri, Sayadi, and Fnaiech ^9^ for expiration dates recognition of products. In pre-processing input image is binarized, skewness of the code is corrected, and the thickness of the touching characters are reduced. Character strings are segmented using a vertical projection technique to extract character images. Individual character images are normalized and convolved with a bank of 2D S-Gabor filters for feature extraction. Feature indexes consisting of the difference between local energy feature maps on subsequent orientation channels, the norm of the difference between subsequent magnitude responses, and the difference between subsequent complex moments magnitude of order one, are used in four filter orientations composing the feature vector used as input for multilayer neural networks for number recognition. The number of output nodes in the neural network is 10 corresponding to the number of digits, the number of nodes in the hidden layer is 50, the learning speed is 0.1, the network learns using the Backpropagation-method, and the number of maximum iterations is set to 5,000 in the method. The achieved average recognition rate reaches 99.3%. The method consistently locates the expiration code of images, and its degraded digit detection rate is high.

The binary large object (BLOB) algorithm with K-nearest neighborhood (KNN) classifier was proposed in Mishra and Jain ^16^ for the recognition of serial numbers printed on labels. Numbers are detected using BLOB-algorithm with filters by color parameters. The KNN classifier used for recognition was first trained with the corresponding numbers. The classifier recognizes detected blobs by comparing them to its trained internal models. The method achieved a detection rate of 88%, and the recognition accuracy of the classifier was 100%. With the inexpensive Linux-based system, the processing speed of the method was 10 frames per second.

In Xiang *et al.* ^18^ a multidirectional illumination and image fusion method for recognition of metal stamping characters on metal surfaces of industrial products was proposed. In the method, the difference in surfaces grayscale values in four source images taken from different lighting directions is used to fuse the images and to eliminate the effect of background brightness differences with enhancing the contrast between the text and the background. Fused images’ character strings are binarized and segmented using the horizontal projection function. Further connected component labeling algorithm is used for single characters separation. For single character histogram of oriented gradients (HOG) -feature extraction, the block of four cells is traversed through the input image in horizontal and vertical directions. Direction and amplitude of the gradient are calculated in each cells in the block. Images’ spatial histogram is obtained by dividing the gradient direction into nine bins in each cell and merging them into a 36-dimensional block feature. By traversing the image, a feature matrix composed of all block features is obtained. The feature vector describing the features of the whole image is obtained by concatenating each row and column of the feature matrix. Backpropagation -neural network is used as a classifier for character recognition. The method achieved a recognition accuracy of 99.6% with an execution time of 2.4 s with a cell size of four pixels with stride three.

### Deep learning methods

A method based on fully convolutional network (FCN) and Tesseract OCR was proposed in Gong *et al.* ^14^ for food packages expiration date detection and recognition. For date region detection Fully Convolutional Network structure decomposing into three parts is used: First branch, the feature extractor stem composing of interleaving convolution and pooling layers is used to extract four levels of feature maps from the input image. Features from different scale levels detects date code regions with different sizes. In the second phase, the feature merging branch, the feature outputs from a different layers of the Feature extractor stem are concatenated, and convolution layer is applied to produce the final feature map. Final feature map is fed into the third branch, the output layer, which contains multiple 1x1 convolution operations to project 32 channels of feature maps into score map, geometry map, and angle map. Score map gives likely-hood that a pixel belongs to the expiry date region, and multi-channel geometry map defines the boundary of the text box, which can be either a rotated box or quadrangle. Network is trained based on the defined loss function using the adaptive moment estimation (ADAM) -optimization tool until performance improves. Location of the final text region area is determined by the locations of the values that are greater than the score map threshold. Geometries associated with these locations on the geometry map are then combined with location-aware non-maximum suppression (NMS) to determine the final text region. Tesseract OCR is used to recognize texts from detected expiry date regions. First, using the maximally Stable Extremal regions (MSER) algorithm, the extracted date code region is binarized with characters being differentiated from the background. Connected component analysis is used to find blobs representing different characters while filtering out small noisy spots. Each candidate blob boundary with the corresponding shape features is extracted for character classification. In the nearest neighbor (NN) classifier blob features are compared to the prototypes representing different characters and classified as the character for which the relative distance is smallest. The proposed system is trained and tested using different types of food package images taken in a natural food store environment. The method achieved a text recognition rate of 98%. However, text recognition errors occurred with blurred characters.

CNN-based deep learning method for water bottle dot matrix characters recognition is described in Muresan, Szabo, and Nedevschi. ^15^ A controlled imaging environment was used for bottle imaging. Transparent and curved plastic bottles are back illuminated, with text area oriented directly to the camera. Mask R-CNN algorithm is used to detect the bottle from the image, returning the bottle shaped bounding box, mask, label, and score of the recognition. The bottle-shaped image is scaled to a predefined size and processed using a morphological gradient operation to outline the objects it contains and further binarized. To find the text area in the image, the white pixels it contains are morphologically processed to form rectangular shaped blobs. By only using the extreme outer contour extraction function, contours are extracted and drawn on a new black image. The properties of the bounding box areas of each contour object are verified, and the original image is cropped using the bounding box coordinates resulting from the included contours. Cropped image with texts in a bounding box is zoomed to twice the size of the original and processed with a sharpening operation before binarization, and morphological processing with the aim of connecting the dots, keeping the characters separate while expanding the number of character pixels. Characters are segmented from the image using vertical and horizontal projections. In post-processing phase often missing dot matrix character parts are reconstructed using morphological dilation. For feature extraction, post-processing phase images, used as features for classification, are equally padded and resized forming a 32x32 pixel square that conforms to CNN’s constraints. Segmented digits are recognized using the LeNet-5 CNN - architecture. Network is trained with a set of ~22500 images in 10 epochs and with batch size of 1000, achieving 97,5% test accuracy.

End-to-end deep learning methods for batch codes recognition printed on cardboard boxes was proposed in Singh *et al.* ^11^ A set of three images is captured at a time in three orientations from a box moving on a conveyor. After pre-processing with motion blur removal of and image sharpening, pre-processed images are subsequently used for the text localization. The connectionist text proposal neural network algorithm is used to detect the text in the image, recognizing the lines of text as a series of fine text proposals. The methods vertical anchoring mechanism predicts the location and text/non-text scores of each fixed-width proposal. The localized text sequences are cropped, and the resulting image is enhanced and adaptively thresholded. Discontinuities in the pixels of the characters are removed before connected components-based contour detection, after which contours with very small width and unexpectedly large height are removed. Each of the contour features are extracted, contours are compared with each other, and groups are formed based on the features belonging within empirically selected values. The objective is to find the character contours for batch codes of a finite length. All characters of the localized text are sent in batches to the capsule-based modified caps net-network, whose structure consists of two feature blocks and two layers of capsules for recognition. Input features extracted by successive convolutions of feature blocks are used to create feature vector, which is then fed to capsule blocks for character prediction. The feature vector enables the network to learn the spatial relationship between features. The achieved recognition accuracy is 85.6% with the real world dataset and 91.3% with the synthetic dataset.

In Ashino and Takeuchi ^10^ the combination of two deep neural networks for dot matrix printing recognition of food drink cartons is proposed. Faster R-CNN, used for expiration date digit location and recognition, first obtains the position and size of characters in an image. The system then scans the expiration date area using a raster scanning method and crops out the area of recognized characters in the image. Character recognition Le-Net network is used for character recognition from the cropped image. The system then combines the results of both neural networks to get a final result based on the spacing of the digits. The limited size of the training data set limited the method's recognition accuracy to 97%, according to the researchers.

The dual DNN method, FCN for text region detection, and convolutional recurrent neural network (CRNN) for text recognition of food packaging is proposed in Gong *et al.* ^7^ Source images of the methods are captured in the real food industry/retail environment, which includes different colors/textures, and low-quality images. A fully connected CNN as in Gong *et al*. ^14^ is used in the text region detection method. Text recognition is performed using CRNN-composed of three parts including the feature extraction part, bidirectional long short-term memory recurrent neural network (LSTM RNN) part, and transcription layer part. In feature extraction, convolution and pooling layers thereof are used to partition the input image into image patches. Feature vectors corresponding to the number of patches are fed to the bidirectional recurrent neural network with the LSTM unit to predict the label distribution. Recurrent layers in Bi-directional LSTM-RNN capture the contextual dependencies between consecutive image patches. Bi-directional LSTM-RNN operates on arbitrary length text sequencies, recognizing texts of different lengths in different formats. The transcription layer of the CRNN converts the predictions of the second LSTM-RNN into a label sequence that maximizes the conditional probability given by the bidirectional LSTM-RNN predictions. Comparing the text recognition performance of the proposed method with Tesseract OCR used to recognize similarly detected texts, the CRNN network performs better in recognizing blurry characters, with OCR being able to misclassify them.

Table 1 summarizes the details of the product code recognition methods analyzed in Section 2, and the recognition rates achieved.

**Table 1.** Summary of product code recognition methods described in Section 2.

No	From	Procedure			Product surface	Recognition accuracy	Dataset
		Extraction method	Segmentation method	Recognition method			
17		Image binarization, detecting the character pixels in image		Comparing the optical patterns consisting of detected pixels to the internal model taught to the system	Consumer products cardboard package	100.0%	Not mentioned
8		Image preprocessing, Binarized Maximum Energy Difference- maps Connected Components- analysis, binarization, morphological processing, characters isolation, normalization, and Gabor Filtering		Gabor-features Classifier	Food package and drink package	98.0%	500 images, 100 samples for each character, in total 2800 samples
9		Image preprocessing, binarization, thinning, and de-skewing	Vertical Pixel Projection	Stretched Gabor features Multilayer Perceptron Network (MLP)	Food package and drink package	99.3%	2000 images, 100 samples for each digits, at all 1000 samples
16		Image pre-processing, intensity-based detection of Binary Large Objects (BLOB) boundaries		KNN-Classifier	Opaque labels flat surface	Detection rate 88.0% Recognition accuracy 100.0%	75 images
18		Fused image preprocessing, binarization, Connected Components labeling and Mathematical Morphology operations	Character strings Horizontal Pixel Projection, Connected Components analysis, preprocessing, and single characters Connected Components labeling	Histogram Analysis using HOG and Back propagation- Neural Network	Metallic liquefied petroleum gas cylinder	99.6%	75-character samples for 37 categories, total 2775 which divided as training and test sets, 1850 characters/training and 925 characters/testing
14		Fully Convolution Deep Neural Network		Tesseract OCR	Food package	98.0%	240 images
15		Mask R-CNN Deep Neural Network, cropped text areas pre-processing and Morphological Processing, Binarization, Morphological Pre-processing, text area contour extraction, text area resizing, and preprocessing	Morphological processing, Vertical- and Horizontal Projection, post-processing, and padding	Modified LeNet-5 Convolutional Deep Neural Network	Plastic water bottle	97,5%	176 images of characters, augmented to 22528 characters
11		Image preprocessing, Connectionist Text Proposal Deep Neural Network based text sequence localization	Text sequence preprocessing, thresholding, Morphological processing, contours feature extraction, similar features-based group formation, and prior knowledge based final contours validation	Modified Caps Net-Deep Neural Network.	Cardboard box moving on a conveyor	91.3%	3700 images
10		Faster R-CNN Deep Neural Network		Le-Net Deep Neural Network	Carton drink packages	97.0%	138 images
7		Fully Convolution Deep Neural Network		Convolution Recurrent Deep Neural Network,	Food package	Detection rate 98.2% Recognition accuracy 95.4%	2424 images

Table 1 summarizes the character extraction and recognition algorithms from various products, and description of their surfaces, recognition accuracies, and training data sets. It can be concluded that machine vision can accurately recognize characters of various shapes printed using various methods on various product surfaces. It should be noted that controlled imaging environments are used during the imaging phase of the product surfaces in. ^11^^,^^15^ Product codes printed on products can now be recognized on surfaces that traditionally normally require human vision using current state-of-the-art methods.

## Results

### Electronic search

**Figure 2.**
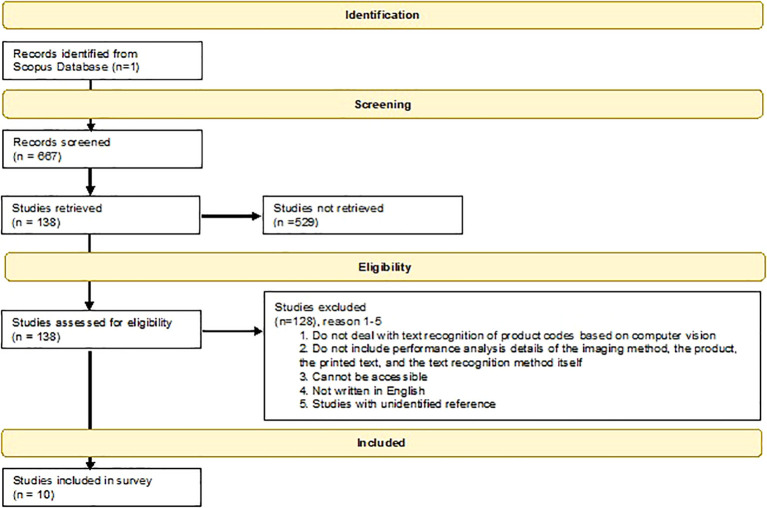
The flow diagram of the article selection process of the current systematic review.

The results are based on a detailed analysis of 10 studies published between 2012 and 2020 which presented state-of-the-art product code recognition methods. ^22^ The PRISMA-based flowchart of this systematic review ^23^ is shown in Figure 2.

### Research question 1: How have machine vision methods capable of recognizing product texts evolved over the last eight years?

In this section, we examine the development of product text recognition methods over the past eight years. Based on the analyzed articles, six relevant aspects can be identified that affect the recognition accuracy:

(1) Changes in the shape, size, position, and placement angle of the packages in the camera view.
(2) Changes in the shape of the packaging surface on which the codes are printed.
(3) Changes in the illumination of the packaging surface.
(4) Low contrast between the text printed on the surface of the product and the background or its variation.
(5) Inconsistencies in the character shapes of the text.
(6) The effect of motion blur caused by the movement of the package.

For the results, the text recognition performance, imaging methods, products, texts printed on them, and printing methods of the articles published during the research period were compared with those of the first year.

Deep learning methods with two consecutive networks are the most tolerant to the most common problems in the field. Despite variations in physical circumstances, changes in the curvature of the package's surface, and changes in illumination, the study's deep learning algorithms recognize the characters. In a controlled imaging environment, they also recognize low-contrast characters, characters with irregular character formats, and images captured from moving packages. Recognition accuracies of the methods are despite these imperfections over 91%.

Conventional recognition methods have evolved to tolerate variations in surface shape and illumination, as well as low contrast between the text and background, during the period of the research. On the other hand, the rate of recognition of regular characters on a plain background has increased. They have a recognition accuracy of more than 99%. Table 2 shows the results of the method development for the most common recognition difficulties in the field.

**Table 2.** Methods tolerance to most common recognition difficulties.

	Publication year	Changes in physical conditions	Changes in the shapes of the packaging surface	Changes in the illumination of the packaging surface	Low contrast between the characters and the packaging background	Irregularities in the shapes of the characters	Motion blur caused by the movement of the packaging
**Deep neural network for text detection and recognition** ^7^^,^^10^	**2020**	✓	✓	✓	✓	✓	✓
**Deep neural network for text detection and recognition** ^11^^,^^15^	**2019**	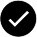	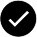	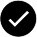	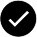	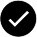	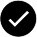
**Deep Neural Network for text area Detection, OCR-recognition** ^14^	**2018**	✓	✓	✓	✓	✗	✗
**HOG feature-based Neural Network Recognition** ^18^	**2018**	✗	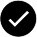	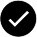	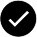	✗	✗
**Detection of Binary Large Objects, KNN- classification** ^16^	**2016**	✗	✗	✗	✗	✗	✗
**Utilization of Gabor filtering** ^8^^,^^9^	**2015**	✗	✓	✓	✓	✗	✗
**Optical Character Recognition** ^22^	**2012**	✗	✗	✗	✗	✗	✗

✓- Exists, 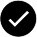
- When imaging in controlled environment, ✗- Is not.

Deep learning has outperformed other recognition methods in the past two years, while no conventional methods have been proposed. The following numbers of deep learning character recognition algorithms have been proposed:

Five of them utilize deep learning to detect text regions. ^7^^,^^10^^,^^11^^,^^14^^,^^15^ Four of them ^7^^,^^10^^,^^11^^,^^15^ use two separate deep networks for text area detection and character recognition. Table 3 represents the number of papers published each year by method of recognition.

**Table 3.** Number of publications by year with recognition methods.

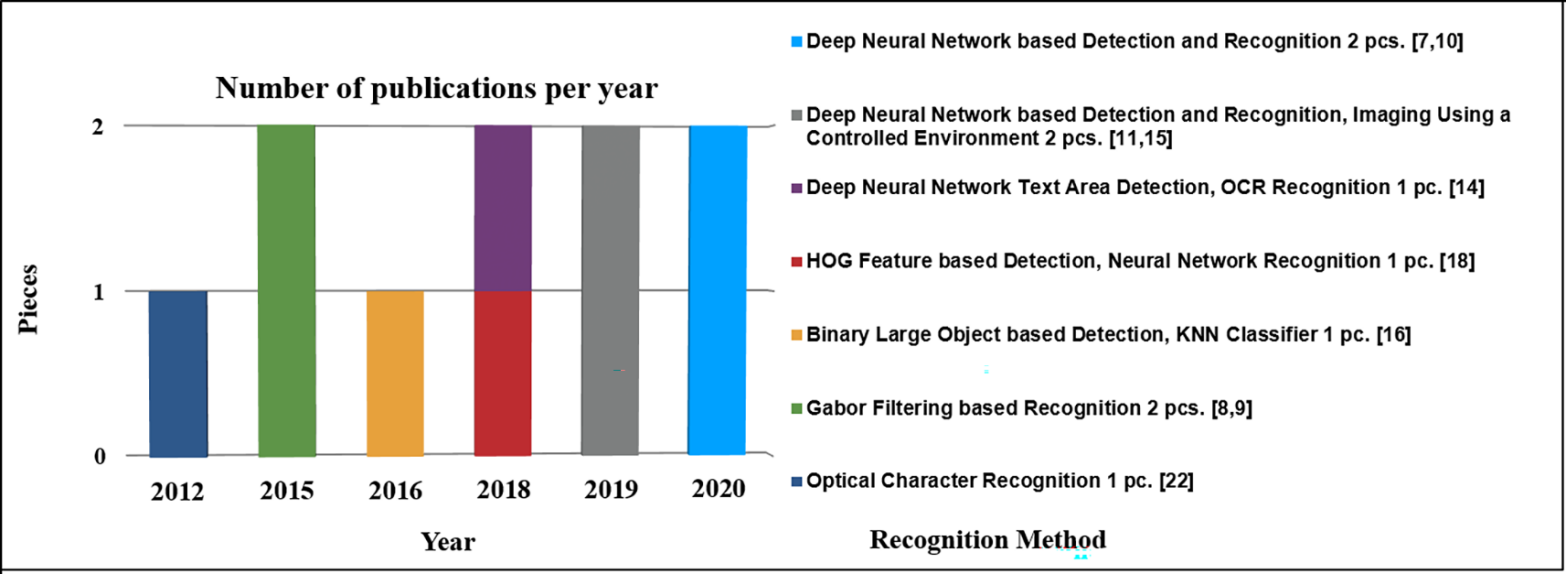

A significant result for this field is the comparison of the performance of conventional character recognition with deep learning when recognizing characters that are inclined, affected by lighting, and printed with low printing quality from food packaging images: The CRNN method has a recognition rate of 95.4%, whereas the Tesseract OCR method has a rate of 31.1%. ^7^

In this research, deep learning is used to recognize characters in a wide range of food packaging, beverage cans, transparent water bottles, and moving boxes. Conventional recognition methods in the study ^8^^,^^9^^,^^16^^,^^18^^,^^21^ included multi-directional illumination of the text area to enhance low-contrast characters, recognition of regularly shaped characters by a computationally efficient BLOB algorithm with the KNN classifier, character recognition based on energy differences in different areas of the image, and the OCR method. The performance of conventional recognition methods has improved in terms of the speed of recognition of clear characters in clear backgrounds, as well as in the recognition of degraded characters.

**Figure 3.**
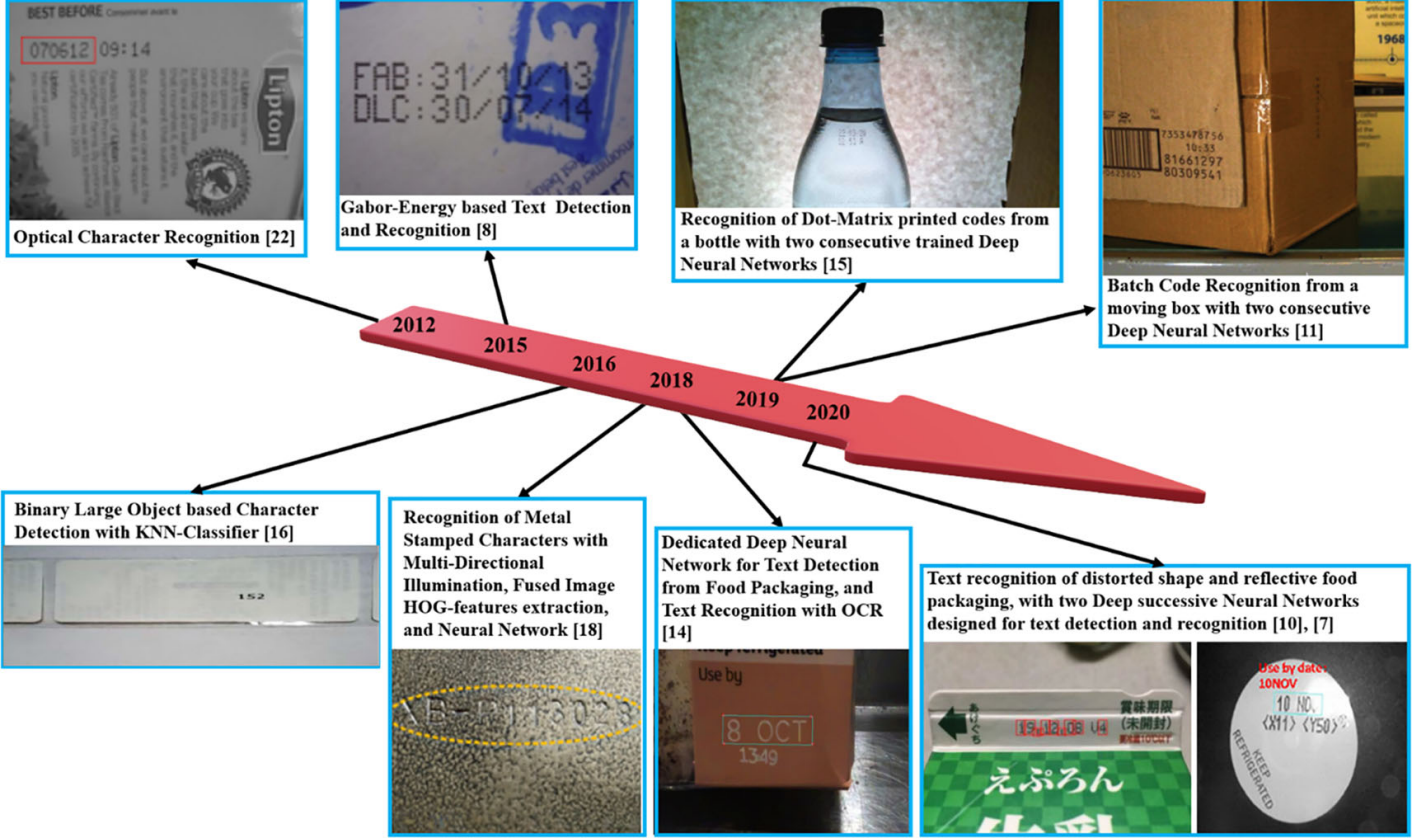
Methods development in their timeline with the source images, characters, and packaging surfaces.

Figure 3 illustrates the evolution of methods in terms of publication years, used source images, character characteristics, and packaging surfaces.

With the set search criteria, ten studies containing performance analysis were found, of which seven proposed methods for expiration date code detection, two for serial numbers, and one for batch code recognition. In addition to them, four research papers from the years 2012-2020 were examined in order to obtain a sufficient knowledge base of the field, they deal with: OCR performance in a variety of environments, ^17^ dot-matrix character segmentation, ^5^ deep neural networks in impaired character recognition, ^13^ and the effect of pre-processing methods in improving general-purpose OCR performance. ^12^

### Research question 2: What are the most common difficulties in recognizing product text?

This section will explore of which are the most common difficulties in recognizing product text. The search strategy used made state-of-the-art product text recognition methods available. Studies of the latest methods include solutions to overcome the most common problems in the field. Analysis of the research material provided answers to the second research question:

The main problems in product code recognition are:

Scene complexity: Variations in physical conditions while capturing images of product packages: Changes in the shape, size, location, and angle of placement of the packages. A natural scene image from package may contain text with arbitrary perspective deformation in a complex background due to its unknown 3D position and orientation.

(1) To solve this problem, Table 4 shows the solutions to the variations caused by physical conditions.

**Table 4.** Position and orientation of the text in the camera scene varies.

Solution:	Implementation method:
Equipment improvement	Using a constrained and controlled imaging environment when acquiring images from the package surface ^5^^,^^15^^,^^18^
	Using a series of three cameras with different orientations to capture images at the same time ^11^
Algorithm improvement	Using a deep neural network models, able to automatically learn effective features for text detection and recognition under variety of scenes ^7^^,^^11^

(2) Due to the change in the shape of the package surface, the intensity of the light varies in different locations, which is reflected as different shades of gray on the same surface, leading to an incorrect recognition result. To solve this problem, Table 5 shows the solutions to alleviate the problem caused by the change in the surfaces shape.

**Table 5.** Solutions to illumination chances in the package surface.

Solution:	Implementation method:
Method improvement	Using multi-directional illumination technology, obtaining a projection image of the target object with different light source directions at fixed points and approximating the three-dimensional structure of the target surface through image fusion technology ^18^
Equipment improvement	Using a constrained and controlled imaging environment when acquiring images from the package surface ^15^
Algorithm improvement	Using a deep neural network models, able to automatically learn effective features for text detection and recognition under variety of scenes ^7^^,^^11^^,^^15^

(3) Low contrast of text printed on the surface of the package. Variations in product label formats. Caused by uneven illumination of a complex package background (not a flat surface). Background colored texts. Complex background with writing style. To solve this problem, Table 6 shows solutions to alleviate the problem caused by the low contrast of the text.

**Table 6.** Solutions to low contrast between text and background in camera view.

Solution:	Implementation method:
Method improvement	Using multi-directional illumination technology, obtaining a projection image of the target object with different light source directions at fixed points and approximating the 3D structure of the target surface through image fusion technology ^18^
Algorithm improvement	Using a deep learning methods for text area detection and text recognition ^7^^,^^15^
	Using image energy-based Gabor-filtering ^8^^,^^9^

(4) Fonts and print style. Inconsistencies in character shapes in manual ink stamping and in dot matrix printed texts. Variations in printing styles such as blurred code due to manual printing, such as ink stamping. ^11^

Some printing methods may produce blurry texts and texts without common features from any other font family. During to the storage duration, texts may be distorted and warped.

Table 7 presents solutions to inconsistency in the shapes of the fonts.

**Table 7.** Solutions to text recognition problems due the irregular fonts.

Solution:	Implementation method:
Algorithm improvement	Using a deep neural network models, able to automatically learn effective features for text detection and recognition under variety of scenes ^7^^,^^10^^,^^11^^,^^15^
	Using a dots connecting algorithm in dot matrix text recognition ^17^
**Solution for font irregularities due to storage duration:**	**Implementation method:**
Method improvement	Image fusion taken in a multi-directional lighting environment for characters HOG- features extraction with neural network classifier ^18^

(5) Motion blur due to movement:

Motion blur caused by acquiring an image of the product package as it moves on the conveyor. To solve this problem, Table 8 shows the solutions to the motion blur.

**Table 8.** Solutions to the motion blur of acquired images of moving packages.

Solution:	Implementation method:
Equipment improvement	Using a controlled imaging environment when acquiring images from the package surface ^5^^,^^11^
Algorithm improvement	Using deep learning methods for text area detection (CTPN) and text recognition (Modified Capsulate Net) ^11^

The answers to the research question have been found through an in-depth literature review of recent research and analysis of the papers included in the research.

## Discussion

This paper provided a detailed literature review of state-of-art product code recognition methods proposed and tested in recent relevant studies. The research questions focused on finding solutions for the development of methods for product text recognition in the last 8 years, and for the most common problems of product text recognition.

Recognition techniques were divided into a previous conventional recognition method period of 6 years, and a deep learning methods period into the last two years. Increased application of deep neural networks for product text recognition since 2018 has made possible to recognize inconsistent characters, detect and recognize text of different sizes in images captured in real-world conditions and recognize text from moving packaging.

Methods with two separate consecutive deep neural networks has made it possible to recognize distorted text and irregular characters on surfaces exposed to light, even in low-quality images. In these methods, deep-learning neural networks have been defined for use in text area detection and in character recognition algorithms. The methods use this structure to extract and learn the features of text regions and characters from a large set of training images, and then recognize the characters in subsequent images using the model they have learnt. Such methods greatly contribute to the recognition of packaging texts in real-life conditions. Conventional recognition methods in the study included multi-directional illumination of the text area to enhance low-contrast characters, recognition of regularly shaped characters by a computationally efficient BLOB algorithm with the KNN classifier, and character recognition based on differences in energy in different areas of the image. The performance of conventional recognition methods has improved in terms of the speed of recognition of clear characters in clear backgrounds, as well as in the recognition of degraded characters.

This study demonstrated that product text recognition techniques have evolved to address the most common research problems presented in the results section of this study.

In the first phase of the study, a comprehensive literature search on state-of-the-art methods was carried out, which was analyzed, and the individual method details were presented with a recognition performance analysis. The results section answers research questions and presents the methods tolerances to the most common difficulties in the field, identified based on analyzes performed on research articles. At the end of the results section, the most common difficulties in this area are presented in detail, together with the proposed solutions from the literature.

This research topic is relatively new, and although studies have been published in this area, there are not many. It would be of particular interest to find further studies where experimental results were obtained with real products and environments. Successful product text recognition also requires consistent thinking when designing the product imaging phase, how to capture an image of each package such that the source image's image analysis can effectively recognize the characters contained within it. Since objects of interest (characters) are imaged with visible light from the surface of the product, the research area itself, the development of recognition algorithms would be facilitated by a well-known standard imaging environment. Similarly, it would be useful for research development to have a data set in which the codes are printed using different printing methods. Such as laser and dot matrix printing, stamping, character pressing, and character pressing with ink marking. In addition, a research topic that deserves attention is the contextual handling of recognized characters. In this field of research, publications often propose solutions for the classification of digits, letters, or combinations thereof. In the packaging handling industry, there is a need to convert a variety of well-defined character sets into electronic form, so research should focus on the contextual understanding of different length codes. With OCR, which has been used for decades, this is done by comparing the result with the grammatical words.

## Data availability

### Underlying data

Open Scientific Framework: Summary of References for Recent advancements in machine vision methods for product code recognition. A systematic review. https://doi.org/10.17605/OSF.IO/8Z54T ^22^

This project contains the following underlying data:

• Summary of References Reviewed for Recent advancements in machine vision methods for product code recognition. A systematic review.xlsx (Summary of references).

Data are available under the terms of the Creative Commons Zero “No rights reserved” data waiver (CC0 1.0 Public domain dedication).

### Reporting guidelines

Open Scientific Framework: PRISMA checklist for ‘Recent advancements in machine vision methods for product code recognition: A systematic review’ https://doi.org/10.17605/OSF.IO/CN42Q ^23^

Data are available under the terms of the Creative Commons Zero “No rights reserved” data waiver (CC0 1.0 Public domain dedication).
