# Supplementary material for: Recent advancements in machine vision methods for product code recognition: A systematic review
Source: F1000Res. 2022 Sep 27;11:1099. [Version 1] doi: 10.12688/f1000research.124796.1 (PMC10521108; doi:10.12688/f1000research.124796.1)
Supplement: Peer review history

# Reviewer report: Reviewer response for version 1

**DOI:** 10.5256/f1000research.137029.r192361

**Published:** 2023-08-22

**Authors:** Zobeir Raisi

**Affiliations:** 1 Electrical Engineering Department, Chabahar Maritime University, Chabahar, Sistan and Baluchestan Province, Iran; 2 University of Waterloo, Waterloo, Ontario, Canada

Upon a thorough review of the manuscript, it is gratifying to note that the paper aligns with the required criteria for acceptance. The authors have demonstrated a comprehensive understanding of the subject matter and have effectively presented their research methodologies and findings. The study's focus on text recognition techniques in the context of pharmaceuticals and perishable foods is well-aligned with the aims and scope of our journal. Including traditional and modern recognition methods and a detailed account of extraction, segmentation techniques, and results showcases a rigorous approach to the research. Furthermore, exploring deep learning techniques and their outperformance in recent years adds valuable insights. Given these strengths, the paper is acceptable for indexing with minor revisions to address some points raised earlier. The authors' work contributes significantly to our understanding of text recognition and its applications and will undoubtedly enrich the scholarly dialogue in this domain.

 To further enhance the strength of the paper, the authors are encouraged to consider the following suggestions:

1. While the authors have concentrated on text instances associated with pharmaceuticals and perishable foods, it is worth acknowledging that benchmark datasets within the scene text detection community encompass many images featuring related content. The authors might consider incorporating recent deep learning methodologies with readily available pre-trained models on platforms like GitHub to augment the research. By evaluating these models on text instance images related to pharmaceuticals and perishable foods, the paper could provide a broader perspective on the performance of the chosen techniques within a more diverse context.
2. To provide a more comprehensive understanding of the applications under study, it is recommended that the authors include qualitative results for the challenges that exist in these types of text instances. This could involve showcasing examples of successful text recognition instances from their research. Additionally, for each application, including detailed captions that specify the encountered challenges would enrich the readers' comprehension. Such qualitative insights can offer a nuanced view of the complexities faced in real-world scenarios, enhancing the overall impact of the paper.
3. Addressing the challenge of occlusion in product text instance spotting and its potential impact on the performance of trained models could enhance the paper's comprehensiveness. Therefore, the inclusion of a discussion regarding this aspect would be beneficial.

Are the rationale for, and objectives of, the Systematic Review clearly stated?

Yes

Is the statistical analysis and its interpretation appropriate?

Yes

Are sufficient details of the methods and analysis provided to allow replication by others?

Partly

Are the conclusions drawn adequately supported by the results presented in the review?

Yes

Reviewer Expertise:

Computer Vision, Deep learning, Text Detection, and Recognition in the Wild.

I confirm that I have read this submission and believe that I have an appropriate level of expertise to confirm that it is of an acceptable scientific standard.

# Reviewer report: Reviewer response for version 1

**DOI:** 10.5256/f1000research.137029.r183263

**Published:** 2023-07-31

**Authors:** Mehwish Leghari

**Affiliations:** 1 Quaid-e-Awam University of Engineering, Science & Technology, Nawabshah, Sindh, Pakistan

This study reviews the text recognition techniques in last eight years particularly from the products related with pharmaceuticals and perishable foods. There were 138 search results returned to the authors from various sources and ten articles were shortlisted for this research.

 Overall the methods used by different researchers have been analyzed and presented systematically in this work. Research works using both the traditional recognition techniques like KNN and modern recognition techniques like deep learning methods have been analyzed. A detailed account of extraction methods, segmentation methods, recognition and results have been presented for all the studies reviewed in this research.

 This research also highlighted that some of the deep learning based techniques use a combination of two networks: one for text area detection and other for character recognition. The research presents a detailed discussion on different problems in text recognition that have been solved in recent years. The solutions to the various problems have been presented from the literature in forms of tables. This research concludes that the deep learning has outperformed the conventional methods in recent years.

 Overall the research work is acceptable though there are two points needed to be clear:

1. Using a deep neural network models, able to automatically learn effective features for text detection and recognition under variety of scenes. (This statements need a justification, that how the use of a deep learning method will solve the said problem i.e. the irregular fonts)
2. The paper claims to deal the text recognition for perishable food and pharmaceutical however it only cited the research using food and food packages. Pharmaceuticals have not been cited in any of the 10 research works.

Are the rationale for, and objectives of, the Systematic Review clearly stated?

Yes

Is the statistical analysis and its interpretation appropriate?

I cannot comment. A qualified statistician is required.

Are sufficient details of the methods and analysis provided to allow replication by others?

Yes

Are the conclusions drawn adequately supported by the results presented in the review?

Yes

Reviewer Expertise:

Pattern Recognition, Fingerprint Recognition, Online Signature Recognition, Machine Learning and Deep Learning.

I confirm that I have read this submission and believe that I have an appropriate level of expertise to confirm that it is of an acceptable scientific standard.
